# Medical School Curricular Changes and Their Impact on Mental Health during the Onset of the COVID-19 Pandemic

**DOI:** 10.1055/s-0044-1795152

**Published:** 2024-12-24

**Authors:** Leen Al Kassab, Laila Fozouni, Christopher Reynolds, Phuong Pham, Valerie Dobiesz

**Affiliations:** 1 Department of Obstetrics and Gynecology, Mass General Brigham, Harvard Medical School, Boston, Massachusetts, United States; 2 Department of Internal Medicine, University of California, San Francisco School of Medicine, San Francisco, California, United States; 3 University of Michigan Medical School, Ann Arbor, Michigan, United States; 4 Department of Emergency Medicine, Brigham and Women's Hospital, Harvard Medical School, Harvard T.H. Chan School of Public Health, Harvard Humanitarian Initiative, Boston, Massachusetts, United States; 5 Department of Emergency Medicine, STRATUS Center for Medical Simulation, Brigham and Women's Hospital, Harvard Medical School, Harvard Humanitarian Initiative, Boston, Massachusetts, United States

**Keywords:** medical education, curricular adaptations, mental health, COVID-19

## Abstract

**Objectives** The main objectives were to identify and categorize the curricular changes that occurred in U.S. medical schools during the onset of the coronavirus disease 2019 (COVID-19) pandemic, and to identify the relationship between curricular changes and COVID-19 surges and student mental health.

**Methods** This Institutional Review Board-approved study consisted of a voluntary online survey of U.S. medical students. A convenience sample of students was reached through Facebook groups, medical student organizations, and administrators. The survey consisted of questions about demographics, curricular changes, and mental health. Univariate and backward stepwise multivariate linear regression were used to assess associations between mental health outcomes and demographic characteristics, curricular changes, and stressors.

**Results** Four-hundred and nine medical students completed the survey from 21 states between May 29, 2020, and August 29, 2020. Seventy-nine percent of respondents reported continuing their basic science curricula virtually. Forty-five percent reported that rotations continued virtually; those reported being offered virtually included internal medicine (77%), family medicine (78%), surgery (70%), obstetrics/gynecology (73%), pediatrics (74%), and psychiatry (76%). The majority of students reported that core clerkships (78%) and subinternships (86%) were not allowed, and away rotations were universally canceled. In univariate linear regression, having in-person subinternships and core clerkships allowed, as well as not relocating for coursework or residence (experienced by 35% of students), was associated with improved mental health outcomes, while experiencing graduation changes (15%) or being from the Southern region was associated with worse outcomes ( *p* < 0.001).

**Conclusion** During the early COVID-19 pandemic, students reported that their medical schools adapted by converting to virtual platforms for basic science and core clerkships. Allowing in-person rotations and limiting relocation were associated with improved mental health outcomes among students. Limitations included sample size, selection bias, and student perceptions.

## Introduction

The coronavirus disease 2019 (COVID-19) pandemic led to unprecedented challenges in health care, with more than 5 million deaths worldwide and nearly universal disruption of in-person activities. 1 In the United States, health care facilities became overwhelmed by the pandemic and recurring surges resulting in over 91 million cases and more than 1 million deaths to date. 2 3 4 The pandemic led to significant societal changes including moving nonessential in-person activities to virtual platforms and delaying educational and other advancement opportunities for youth globally.

Historically, a wide range of disasters have disrupted medical education including man-made disasters such as World Wars, natural disasters such as Hurricane Katrina, and biological disasters. 5 The 1918 Spanish flu resulted in school closures around the United States, 6 at a time when limited diagnostic testing, treatment modalities, and other viral countermeasures existed. 7 Schools able to stay open did so by implementing contact tracing and mandatory quarantining of sick students. 8 To manage the severe adult respiratory syndrome outbreak in the early 2000s, medical schools implemented new solutions to ensure student advancement. Many schools replaced clinical patient encounters with videotaped vignettes, student volunteers, mannequin simulators, and telephone-based patient services in which students could participate without risking infection. 9 During both the 2009 H1N1 virus outbreak and the 2014–2016 Ebola virus outbreak in West Africa, medical education was suspended or delayed 10 to limit community spread, protect vulnerable students, and cope with staff shortages. 9 10 11 12 However, innovative solutions, including e-learning, were not successfully implemented during these outbreaks. 13 Disasters are projected to accelerate in the future due to climate change, resource scarcity, and armed conflicts, and it is vital that medical schools develop curricular plans to mitigate the impact of these events on medical student education and well-being.

There remains uncertainty on best practices to implement during disasters such as pandemics for adapting medical school curricula. During the COVID-19 pandemic, medical students across the United States demonstrated a desire to help patients by volunteering in call centers, creating patient care materials, and helping with grocery shopping. 14 Many called for formally integrating medical students into the public health system to increase workforce capacity, contact tracing, testing abilities, and vaccine distribution. 15 The role of medical students during the onset of the pandemic was debated, with some arguing that students should be treated as “junior doctors” and integral members of the health care team, while others asserted that trainee participation was not worth the risk of infection or utilizing scarce health care resources. 9 14

While the COVID-19 pandemic interrupted medical education on an unprecedented scale, there is a paucity of data on curricular adaptations in medical education during the pandemic and their impact on students' mental health. This study aimed to identify and categorize the curricular changes that occurred in the U.S. medical schools during the onset of the COVID-19 pandemic, and to identify the relationship between curricular changes and COVID-19 surges and student mental health.

## Methods

### Study Design and Sampling

This Institutional Review Board (IRB)-approved study consisted of a single voluntary online survey of allopathic medical students across the United States ( Appendix available online). The survey was anonymous using deidentified data and obtained after written informed consent. A distribution list was created including all (154) U.S. medical schools from the American Medical College Application Service database. Medical students in the United States were invited to participate through two methods: Facebook messages to medical student administrators of medical school Facebook class groups and emails to medical student organizations, including the American Medical Association medical student branch, and medical school administrators such as Deans of Student Affairs found on individual medical school websites. The study was conducted from May 29, 2020, to August 29, 2020.

Respondents were asked demographic questions including their school's state location and whether in clinical or preclinical years of medical training.

### Survey Content and Administration

The survey link was sent to key contacts on the described distribution lists via email and Facebook messages, with a request to disseminate the survey to the school or group's student listservs across all student years.

Kobo Toolbox, 16 a free and open-source data collection, analysis, and management resource used globally for research in humanitarian settings, was the survey platform used. The survey instrument was created by study authors adapting a previously used survey tool by one of the authors (P.P.).

#### Curricular Changes

Respondents were asked questions regarding the impact of the pandemic on their training, including the stage in the school's medical curriculum, how curricula were continued, the need to relocate for classes, the ability to attend classes and participate in clinical rotations, which clinical rotations were being offered and in what format at all levels, and impact on graduation plans.

#### Mental Health Outcomes

The survey also asked questions regarding the impact of the pandemic on how well-equipped, prepared, and interested students were in participating in clinical rotations and volunteer opportunities, as well as on any challenges or stressors experienced. The survey included the Patient Health Questionnaire-4 for depression and anxiety, 17 a validated tool for self-reported measures of depression and anxiety. This was used to evaluate respondents' mental health and scored on a Likert scale of 0 (not at all) to 4 (nearly every day). Responses were compiled to create a mental health score ranging from 0 to 16, with higher scores indicating increased mental distress.

### Data Analysis

Demographic data were presented as the total number of respondents and frequencies. Univariate and backward stepwise multivariate linear regression were used on Stata (v15, SE) to assess associations between mental health variables and demographic characteristics, curricular changes, and stressors.

The correlation with COVID-19 surges in each state was calculated by inputting data collection time from May to August 29, 2020, into the Brown School of Public Health risk levels dashboard. 18 A surge was defined by the doubling of the 7-day moving average of a number of cases per 100,000 people, sustained for more than a 7-day period, with a minimum of 10 cases per 100,000 people (10 being the doubling of 5 cases per 100,000 as defined by the Centers for Disease Control and Prevention as the cutoff for level 2 moderate-risk state during the study period). The dashboard graphs per state were used to measure if a surge occurred in each state of interest before (3/25–5/29) and during the defined study duration (5/29–8/29). ( Appendix for illustration).

## Results

### Baseline Characteristics

During the 3-month study period, 409 medical students took the survey, out of 92,626 nationally, 19 not all of whom were reached, from 21 states across all four Association of American Medical Colleges (AAMC) regions (Western, Northeastern, Southern, and Central). Sixty-eight percent identified as female, 65% were in their preclinical years, and 34% were in their clinical years of medical school. Baseline characteristics are shown in Table 1 .

**Table 1:** U.S. medical student demographics by region

Demographic and impact		Total	Region				*p* -Value
			Western ( *N* = 67)	Northeastern ( *N* = 158)	Southern ( *N* = 155)	Central ( *N* = 22)	
Sex	Female identifying	276 (68%)	47 (70%)	112 (71%)	97 (63%)	16 (73%)	0.21
	Nonbinary	3 (1%)	0	3 (2%)	0	0	
Continuing basic science	In-person	2 (1%)	0	0	2 (1%)	0	0.005
	Combination	76 (19%)	7 (10%)	20 (13%)	44 (28%)	5 (23%)	
	Virtually	321 (79%)	60 (90%)	134 (85%)	108 (70%)	16 (73%)	
	No	0	0	0	0	0	
	Other	5 (1%)	0	3 (2%)	1 (1%)	1 (5%)	
Relocated	Yes, moved residence	142 (35%)	19 (28%)	59 (38%)	52 (34%)	11 (50%)	0.26
	Yes, coursework relocated	7 (2%)	0	3 (2%)	3 (2%)	0	0.63
	No remained in residence	267 (65%)	48 (72%)	100 (64%)	105 (68%)	13 (59%)	0.58
Rotations	Virtual	150 (45%)	31 (21%)	41 (28%)	61 (41%)	15 (10%)	<0.001
	Core clerkships allowed	73 (22%)	17 (24%)	24 (33%)	23 (32%)	8 (11%)	0.019
	Subinternship allowed	47 (14%)	16 (34%)	12 (26%)	12 (25%)	7 (15%)	<0.001
	Away rotations allowed	4 (1%)	3 (5%)	0	0	1 (6%)	0.003
	Nonhospital rotations allowed	165 (50%)	22 (40%)	61 (49%)	77 (58%)	4 (22%)	0.01
	Volunteer rotations allowed	51 (15%)	7 (13%)	30 (24)	12 (9%)	2 (11)	0.008
Graduation change	Graduating earlier than expected	3 (1%)	0	3 (2%)	0	0	0.66
	Graduating later than expected	6 (1%)	1 (1%)	3 (2%)	2 (1%)	0	
	Uncertain	63 (16%)	8 (12%)	24 (15%)	28 (18%)	3 (14%)	
	No	330 (82%)	58 (87%)	125 (81%)	125 (81%)	19 (86%	
Participate in-person rotations, *n* = 138	Yes	58 (42%)	18 (62%)	16 (29%)	15 (27%)	7 (13%)	0.001

### Curricular Adaptations

Respondents were asked about their medical school's adaptations at the time of survey completion. Among all participants, the majority (79%) indicated that the preclinical basic science curriculum was virtual or a combination of virtual and in-person (19%).

When asked if their medical school allowed clinical rotations ( *n* = 335), half of the participants indicated they were completely removed from the hospital (50%) or indicated they were allowed to participate in clinical rotations virtually (45%). A smaller percentage (23%) reported they were allowed to continue in-person core clerkships ( Table 2 ). The clinical rotations being offered virtually ( *n* = 113) included family medicine (78%), internal medicine (77%), psychiatry (76%), and obstetrics/gynecology (73%) ( Table 2 ).

**Table 2:** Clinical rotations offered

	Virtual	In-person		
	%, *n* = 113	Core clerkship %, *n* = 58	Subinternship %, *n* = 41	Away, *n* = 3
Internal medicine + subspecialties	77	91	90	1
Family medicine	78	90	66	1
Surgery + subspecialties	70	88	73	1
Obstetrics and gynecology	73	91	63	1
Pediatrics	74	81	75	1
Neurology	69	81	63	0
Psychiatry	76	79	63	1
Radiology	50	50	51	2
Emergency medicine	45	65.5	56	2
Other	12	7	12	2
	Electives: public health/biostatistics	Pulled out mid-March–June 1	Pulled out mid-March–June 1	If a home medical school program without a specialty
	Virtual ICU/ID COVID-19/Path/Crit care/PM&R/anesthesia	Unsure	Unsure	

Abbreviations: COVID-19, coronavirus disease 2019; Crit care, critical care; ICU, intensive care unit; ID, infectious disease; Path, pathology; PM&R, physical medicine and rehabilitation.

Respondents reported that core clerkships rotations offered in person ( *n* = 58) included major core rotations such as internal medicine (91%), obstetrics/gynecology (91%), family medicine (90%), surgery (88%), pediatrics (81%), neurology (81%), and psychiatry (79%). In contrast, fewer subinternships were reported to be offered in person ( *n* = 58), while 90% reported internal medicine subinternships, and 75% or less reported other core rotations ( Table 2 ). In-person away rotations were nearly eliminated with only 1% ( *n* = 3) reporting that their medical schools allowed away rotations. Per respondents, this exception occurred only if the medical school programs did not have the specialty available as a rotation.

Among students in their clinical years, 42% ( *n* = 139) participated in in-person rotations, whereas 70% reported an interest in participating in clinical rotations. Thirty-five percent of students had to relocate and move residence for virtual classes, while 2% had their coursework relocated ( Table 3 ). Eighty-two percent of students did not experience any changes to graduation plans, 15.5% of students were uncertain about changes to graduation due to COVID-19, 1.6% graduated later than expected, and 0.7% graduated earlier than expected due to the pandemic.

**Table 3:** Multivariate linear regression with mental health symptoms as an outcome of interest among U.S. medical students during the COVID-19 pandemic, May 29 to August 29, 2020

Variable		Nervous		Depressed		Worrying		Low interest		Mental health score	
Coefficient											
*p* -Value		Uni	Multi	Uni	Multi	Uni	Multi	Uni	Multi	Uni	Multi
Sex		0.12		0.02		−9		−0.01		0.23	
		−0.25		0.89		0.41		0.93		0.527	
Continuing basic science	Fully virtual	0.01		−0.15		−0.01		−0.05		−0.21	
		0.92		0.24		0.93		0.65		−0.63	
Relocated	No	−0.18		− **0.23**		− **0.23**		− **0.24**		− **0.89**	
		0.08		**0.03**		**0.04**		**0.02**		**0.016**	
Graduation change		**0.49**		**0.49**		**0.5**		**0.37**		1.86	
		**<0.01**		**<0.01**		**<0.01**		**<0.01**		**<0.001**	
In-person rotations		− **0.5**		− **0.55**	− **0.86**	−0.34		− **0.39**		−1.78	
		**0**		**<0.01**	**0.02**	0.06		**0.03**		**0.004**	
Rotations allowed	Yes virtual	−0.11		−0.2		−0.14		−0.21		−0.65	
		0.35		0.08		0.25		0.06		0.1	
	In-person core clerkships	− **0.31**	Omitted	− **0.29**	Omitted	−0.24		−0.14		− **0.97**	Omitted
		**0.02**		**0.04**		0.1		0.31		**0.042**	
	In-person subinternship	− **0.4**		− **0.45**	− **1.25**	−0.28	− **0.62**	− **0.29**	− **0.29**	− **1.41**	− **1.97**
		**0.01**		**<0.01**	**<0.01**	0.11	**0.004**	**0.06**	**0.06**	**0.012**	**0.015**
	Away rotations	− **0.88**		−0.53		−0.39		0.16		−1.64	
		**0.09**		0.32		0.49		0.76		0.37	
	Nonhospital rotations	0.11		0.21		0.04		0.14		0.49	
		0.32		0.08		0.72		0.21		0.21	
	Volunteer rotations	−0.11		−0.17		−0.13		−0.5		−0.48	
		0.48		0.3		0.43		0.73		0.38	
Rotations virtually	Internal medicine	−0.19		− **0.49**		− **0.53**		−0.24		−1.46	
		0.41		**0.03**		**0.02**		0.24		0.054	
	Family medicine	−0.12		−0.3		−0.34		−0.03		−0.74	
		0.61		0.28		0.15		0.9		0.34	
	Surgery	−0.29		− **0.48**		− **0.54**	− **0.57**	−0.3		− **1.62**	
		0.18		**0.02**		**0.01**	**0.006**	0.1		**0.02**	
	Obstetrics and gynecology	−0.23		−0.32		− **0.44**		−0.1		−1.09	
		0.29		0.13		**0.05**		0.59		0.126	
	Pediatrics	0.01		−0.19		−0.26		−0.09		−0.54	
		0.98		0.38		0.24		0.64		0.46	
	Neurology	−0.14		−0.19		−0.16		0.02		−0.47	
		0.51		0.36		0.45		0.92		0.5	
	Psychiatry	−0.16		−0.29		−0.42		−0.1		−0.98	
		0.48		0.19		0.07		0.61		0.193	
	Radiology	−0.17		0.14		−0.12		0.22		0.07	
		0.4		0.45		0.53		0.2		0.91	
	Emergency medicine	−0.17		−0.12		−0.06		−0.02		−0.37	
		0.3		0.52		−0.77		0.89		0.565	
	Other	−0.04		0.17		−0.03		0.17		0.31	
		0.88		0.57		0.92		0.54		0.755	
Core rotations in-person	Internal medicine	−0.35		−0.79	− **2.32**	−0.67		− **1.03**		− **2.8**	− **3.18**
		0.44		0.06	**<0.01**	0.11		**0.01**		**0.04**	**0.019**
	Family medicine	0.32		0.01		−0.24		−0.19		−0.1	
		0.44		0.97		0.55		0.62		0.939	
	Surgery	0		−0.53		− **0.73**		−0.55		−1.8	
		1		0.15		**0.04**		0.12		0.136	
	Obstetrics and gynecology	−0.13		−0.79	**1.42**	−0.67		− **0.81**		−2.4	
		0.78		0.06	**0.04**	0.11		**0.05**		0.088	
	Pediatrics	0.02		−0.17		−0.15		−0.31		−0.62	
		0.96		0.57		0.62		0.3		0.545	
	Neurology	0.13		0.28		0.3		0.14		0.85	
		0.69		0.36		0.33		0.63		0.41	
	Psychiatry	−0.38		− **0.62**		−0.17		− **0.64**		− **1.81**	
		0.22		**0.03**		0.58		**0.02**		**0.06**	
	Radiology	0.14		0		−0.29		−0.11		0.32	
		0.58		1		0.24		0.65		0.69	
	Emergency medicine	−0.02		0.04		0.13		−0.45		−0.29	
		0.93		0.86		0.6		0.07		0.73	
	Other	− **1.1**	− **1.11**	−0.46		−0.31		0.1		−1.79	
		**0.02**	**0.02**	0.33		0.52		0.84		0.26	
Subinternship rotations in-person	Internal medicine	−0.75		− **0.87**		− **1.18**		− **0.93**		− **3.73**	
		0.09		**0.03**		**<0.01**		**0.02**		**0.005**	
		0.19		0.43		0.16		0.97		0.26	
Region (vs. West)	Northeastern	0.03		0.22		0.04		0.1		0.39	
		0.86		0.15		0.77		0.47		0.76	
	Southern	0.18		**0.42**		0.28		0.22		**1.09**	
		0.23		**<0.01**		0.07		0.13		**0.034**	
	Central	0.13		0.36		0.32		0.13		0.95	
		0.57		0.16		0.22		0.58		9.27	
Surge before survey		− **0.29**		− **0.21**		−0.19		− **0.19**		− **0.88**	
		**0.01**		**0.04**		0.07		**0.05**		**0.013**	
Surge during survey		0.08		0.07		0.14		0.04		0.33	
		0.44		0.49		0.19		0.69		0.35	

Abbreviation: COVID-19, coronavirus disease 2019.

Bold numbers have a *p* value < 0.05, indicating significant value.

### Mental Health Associations

We broadly assessed demographic and curricular factors and their association with mental health outcomes in univariate and multivariate regression models. In univariate models, we found no association between sex, continuing basic science curriculum, and reporting virtual, nonhospital, and volunteer rotations with any of the five mental health outcomes.

#### Factors Associated with Improved Mental Health Outcomes

Regarding positive impacts on mental health outcomes, not relocating was associated with decreased depression (−0.23, *p* = 0.03), worrying (−0.23, *p* = 0.04), low interest (−0.24, *p* = 0.02), and mental health score (−0.89, *p* = 0.02) in univariate regression. Reporting in-person rotations was associated with decreased nervousness (−0.5, *p* = 0), depression (−0.55, *p* < 0.01), low interest (0.37, *p* < 0.01), and mental health scores (−1.78, *p* < 0.01). Reporting in-person core clerkships was associated with decreased nervousness (−0.31, *p* = 0.02), depression (−0.29, *p* = 0.04), and overall mental health score (−0.97, *p* = 0.04), while in-person subinternships were associated with decreased nervousness (−0.4, *p* = 0.01), depression (−0.45, *p* < 0.01), low interest (−0.29, *p* = 0.06), and mental health score (−1.41, *p* = 0.01). Various rotations being offered either virtually or in person, ranging from internal medicine, surgery, and obstetrics and gynecology, were also associated with reduced mental health scores ( Table 3 ). Finally, experiencing a COVID-19 surge prior to the survey was associated with decreased nervousness (−0.29, *p* = 0.01), depression (−0.21, *p* = 0.04), low interest (−0.19, *p* = 0.05), and mental health score (−0.88, *p* < 0.01).

#### Factors Associated with Worsened Mental Health Outcomes

Experiencing any changes to graduation dates was associated with worsened nervousness (0.49, *p* < 0.01), depression (0.49, *p* < 0.01), worrying (0.5, *p* < 0.01), low interest (0.37, *p* < 0.01), and total mental health score (1.86, *p* < 0.001) in univariate regression models ( Table 3 ). Enrollment in a medical school in a Southern state, compared with a Western one, was associated with increased depression (0.42, *p* = 0.01) and total mental health score (1.09, *p* ≤ 0.01).

#### Multivariate Regression

In multivariate linear regression run using backward stepwise regression with depression as the primary outcome, reporting in-person rotations remained associated with a 0.86-point decrease in depression score ( *p* = 0.02), in-person subinternships with a 1.25-point decrease ( *p* < 0.01), and in-person core internal medicine with a 2.32-point decrease ( *p* < 0.01). With low interest as the primary outcome, reporting in-person subinternships was associated with a 0.29-point decrease in the low interest score. In a multivariate model with mental health score as the primary outcome, reporting in-person subinternships was associated with a 1.97-point decrease in mental health score, while reporting in-person core internal medicine rotations was associated with a 3.18-point decrease. Finally, in multivariate linear regression run using backward stepwise regression, only experiencing “other” in-person rotations remained associated with decreased nervousness score (−1.11, *p* = 0.02) ( Table 3 ).

## Discussion

### Curricular Changes Due to COVID-19

At the onset of the COVID-19 pandemic, rapidly evolving changes to educational curricula were made to address infection control concerns, impacting medical and other health professional students.

The COVID-19 pandemic served as a catalyst for medical education transformation including students taking the opportunity to volunteer and support public health responses, educating the public, and community building, adapting curricula in real time and innovating new methods of instruction and testing, and graduating some students early to join the health care task force. 20

The structure of most medical school curricula in the United States entails a preclerkship phase to build a foundation of medical knowledge, a clerkship phase that entails core, required clinical rotations in the hospital and other clinical settings, and a postclerkship phase that involves advanced electives entitled “subinternships.” There may also be classroom-based or clinical electives that are optional based on student interests.

Medical education in the United States covers a highly structured curriculum in a variety of preclinical and clinical environments whose architecture and requirements are set by the Liaison Committee for Medical Education (LCME) and the Accreditation Council for Graduate Medical Education. 21

The COVID-19 pandemic posed significant disruptions to clinical (hospital-based patient care) and didactic (classroom-based content lectures) learning environments. Transitions to remote learning and modified clinical experiences often led to the suspension of in-person classes with a transition to virtual lectures, shortened clinical rotations, and cancellation of domestic and international away clinical electives (an optional rotation at any institution other than the home institution). 14 22 23 The adaptation guidelines recommended during our study period among medical schools to clinical clerkships are not well documented; the most restrictive was a guideline in March 2020 from the AAMC calling to remove students from clerkships. Many schools demonstrated innovative approaches to the COVID-19 pandemic including rearranging vacation time around surges, using postponed clerkship rotations to teach students about social medicine, public health, medical ethics, and other topics often less emphasized in medical education, as well as using real-life epidemiological studies of COVID-19 to teach public health and disease transmission. 24

Our study was conducted in the early stages of the pandemic when the greatest uncertainty existed, and the role of medical students was being debated. It validates previous literature describing the shift to virtual platforms, 9 13 14 15 with our study participants reporting that 79% of preclinical basic science curricula and 45% of clinical rotations became virtual. We further delineated curricular adaptations and evaluated the impacts of these changes on students' mental health during this time of dynamic curricular changes. Our study identified protective factors for mental health, including preserving in-person core clerkships and subinternships, as well as factors that were associated with worse mental health, including experiencing changes to graduation dates and being from the South.

The AAMC data of COVID-19 pandemic curricular changes captured by the LCME annual medical school questionnaire that overlapped our data collection time frame (March 2020 to June 2021) showed that 97 of 155 (63%) medical schools decreased the length of one or more required clerkships, 142 of 155 (92%) replaced at least some in-person preclerkship clinical skills teaching sessions with virtual sessions and 149 of 155 (96%) replaced at least some in-person small group sessions with virtual ones. In addition, 139 of 155 (90%) schools replaced at least some in-person clinical encounters with virtual/telemedicine, and 149 out of 155 (96%) replaced at least some in-person didactic sessions with virtual sessions. 25

Medical schools such as the University of California in San Francisco and Northwestern University describe similar curricular adaptations to our study findings. Northwestern University similarly temporarily removed students from direct patient care, migrated the preclinical curriculum online, offered virtual clerkships and clinical assessments, and created new online electives. Students also engaged in community service. 21

A cross-sectional survey of 65 fourth-year medical students in Buffalo, New York, indicated that surgery, internal medicine, and obstetrics and gynecology were the top specialties impacted by the COVID-19 pandemic. Students appreciated the implemented tele-education curricula and case-based video learning with abbreviated clinical exposure on resumption of rotations though most did not find it as effective. 26

Similar adaptations were also done internationally, including in Saudi Arabia, where the transition to online curricula is described. 27 In Korea, a survey of medical school deans also illustrated adaptations of brief suspensions of in-patient care followed by moving to online curricula and integrating technological advances into medical education that was viewed positively. 28

There are both advantages and limitations to changes that transition to virtual learning. 29

In the setting of transitioning clinical skills and practical procedure training to being online, access to the internet, technology, and computer education posed resource allocation challenges in developing countries and further widened the disparities in medical education. 30

#### Impact of COVID-19 on Student Mental Health

The COVID-19 pandemic exacerbated mental health stressors for health care workers. The U.S. medical students have a baseline prevalence rate of anxiety at 11%, 31 32 33 34 and depressive symptoms are known to increase as clinical years progress; first-, second-, third-, and final-year students experience depression rates of 14, 23, 30, and 14%, respectively. 35 The impact of curricular adaptations during the early stages of the pandemic on mental health stressors for medical students remains understudied.

Health care providers are particularly vulnerable to emotional distress during pandemics, given similarly captured concerns for their risk of exposure to the virus, concern about infecting and caring for their loved ones, shortages of personal protective equipment, longer work hours, and involvement in emotionally and ethically fraught resource-allocation decisions.

Numerous emotional outcomes, including stress, depression, irritability, insomnia, fear, confusion, anger, frustration, boredom, and stigma were documented associated with quarantine, some of which persisted after the quarantine was lifted. 36

In a survey of 1,027 Indonesian medical students in July 2020 using a Likert scale to assess mental health outcomes, 44.6% of participants had stress, 47.8% had anxiety, and 18.6% had depression. It also similarly found that the zonation of transmission risk of COVID-19 infection being higher plays a significant role in a higher score for fear associated with COVID-19. 37

In a review of 13 studies evaluating the impact of COVID-19 on medical student curricula and mental health, it was found that per American Medical Association guidelines, after a temporary pause to all clinical rotations, in-person curricula moved to virtual modalities. This limited students' ability to explore their specialties of interest and confidence in competency. There was also the report of higher levels of anxiety, stress, and exhaustion, with higher levels in female as compared with male students. 38

Another survey of 741 U.S. medical students evaluated students' anxiety with a 7-point Likert scale. There was a statistically significant increase in self-reported emotional exhaustion and burnout from before the pandemic and after the pandemic started ( *p* < 0.001). 39

A longitudinal study of 217 Indian undergraduate medical students who completed the Depression Anxiety Stress Scale 21 items before and during COVID-19 revealed a significant increase in both prevalence and levels of anxiety and stress during this period ( *p* < 0.001); however, levels of depression did not change. 40

Another study of 549 Moroccan medical students found that 341 (62.3%), 410 (74.6%), 344 (62.6%), and 379 (69%) students, respectively, self-reported anxiety, depression, insomnia, and distress during the early stages of the pandemic. 41

In a cross-sectional study during the height of the COVID-19 pandemic, 960 medical students were surveyed finding 25.1% ( *n* = 241) screened positive for depression, 40.4% ( *n* = 388) screened positive for anxiety, 21.3% ( *n* = 201) met criteria for at least one dimension of burnout, 19.0% ( *n* = 182) started or increased substance use, and 7.2% ( *n* = 69) experienced thoughts of self-harm or suicide. Rates of anxiety and substance use among medical students were higher than previously reported, and rates of burnout and thoughts of self-harm or suicide were found to be lower. These results indicated that aspects of remote learning integrated during the pandemic could be protective but need further study. 42

We found that reported in-person core clerkships and sub-internships were associated with improved mental health scores for students. This protective effect may be due to minimizing disruptions to training, mitigating anxiety about career preparedness, and fulfilling many students' interests in contributing to the health care workforce during the pandemic.

We also found that reporting the need to relocate or experiencing changes in graduation dates was associated with worsened mental health scores and should be considered an important factor to be mitigated during disaster planning. Given the increased burden of mental health stressors identified, academic programs should consider the potential negative psychological impacts during crises situations on students and develop preparedness and adaptation strategies that allow for minimal disruptions in medical curricula and education and provide additional support services for students. Limiting the relocation of coursework and students if possible and allowing students to continue their subinternships and core clerkship rotations appear to mitigate the adverse impacts.

#### Recommendations

Further research is needed to validate these findings and explore the long-term impact on student performance outcome measures due to conversion to virtual or hybrid formats. This data can help inform future medical schools' response to pandemics and other disasters and is a ripe area for further research given the rise in global and climate-related disasters. As disasters are predicted to increase exponentially, developing evidence-based guidelines regarding best practices can improve disaster preparedness for academic medical institutions and mitigate medical education disruptions and the negative mental health impacts for trainees.

## Limitations

Our study has several limitations. First, given there is no publicly available database of U.S. medical students, we relied on emailing school administrators and Facebook class group administrators for survey distribution. Not all of those who were reached responded, and we were unable to assess whether the surveys had been successfully distributed to students at all schools; therefore, the response rate was unable to be measured. We did receive responses from 21 states across all 4 AAMC regions. Since responses were deidentified, and some states have only one medical school, only U.S. regions of medical schools were reported, rather than school names or states. Furthermore, the survey collected individual student perceptions rather than institutional data. This includes questions regarding clinical rotations, which students may not have known about at their institution other than the one(s) they personally experienced when completing the survey. In addition, to quickly roll out this study in the early stages of the pandemic, we relied on a voluntary survey which may result in nonresponse and selection bias, as those who responded to the survey may differ from the rest of the medical student population. Despite these limitations, we believe the data gathered during the outset of the pandemic provide invaluable information regarding the medical curricula adaptations and the impact they had on medical student mental health.

## Conclusion

Adaptations to U.S. medical school curricula primarily involved converting to virtual platforms for the basic sciences and virtual and/or hybrid core clerkships with away rotations not being allowed. Most core clerkships were offered virtually or in-person, with less offered in radiology and emergency medicine. Allowing subinternships and clerkship rotations and limiting relocation mitigated the negative mental health impacts on medical students at the COVID-19 pandemic onset. Understanding the protective factors associated with curricular changes on medical student mental health outcomes may help inform future disaster preparedness. The long-term impacts of curricular adaptations should be further investigated as disasters (biological, man-made, and environmental) are projected to continue in the future.
