# Supplementary material for: Medical School Curricular Changes and Their Impact on Mental Health during the Onset of the COVID-19 Pandemic
Source: Avicenna J Med. 2024 Dec 24;14(4):194–203. doi: 10.1055/s-0044-1795152 (PMC11896715; doi:10.1055/s-0044-1795152)
Supplement: Supplementary file 1 — Supplementary Material [file 10-1055-s-0044-1795152-s240056.pdf]

COVID-19 Medical Student Survey

HARVARD HUMANITARIAN INITIATIVE

Why is this survey being done?
This study is being done by Brigham and Women's Hospital and Harvard Humanitarian Initiative. This study is self-funded. The purpose of this research is to analyze the current medical response to COVID-19. The research will be used to inform response efforts.

Participation is voluntary
We are asking you to participate voluntarily in this study. It is your choice whether to participate or not. If you choose to participate, you may change your mind and exit the study at any time.

How long will it take?
Completing the survey should take about 3-5 minutes.

Risks and Possible Discomforts
Taking part in the survey may make you anxious or worried.

Benefits of Participation

There are no direct benefits to you from taking part in this study, others may benefit from what we learn. You will not be paid for participating.

Privacy and Confidentiality

This is an anonymous survey. Neither phone numbers, email addresses, URL addresses, nor IP addresses will be recorded or retained in any way. We are an independent academic research group and not affiliated with any government.

If you have any questions, concerns or complaints about this study
you may contact Dr. Phuong Pham, Principal Investigator, via email at phuong.pham@kobotoolbox.org any time.

Are you a medical student? *

- (●) Yes
- ( ) No

Do you agree to take part in this survey? *

- (●) Yes
- ( ) No

Describe your stage in your school's medical curriculum prior to the coronavirus outbreak?

- ( ) Preclinical
- (x) Clinical (Rotations, Sub-internships, Electives, etc)

During the COVID pandemic, is your medical school continuing your basic science curriculum?

- ( ) Yes; In-person
- (x) Yes; Virtually
- ( ) Yes; Combination of in-person/virtual
- ( ) No; Unable to continue
- ( ) Other

If other, please describe:

Have you relocated to continue your medical education? (Check all that apply)

- [ ] No; Remained in current setting
- [x] Yes; Moved residence for virtual classes
- [ ] Yes; Medical school's coursework or classes had to relocate to other facility/location

Has your medical school allowed clinical rotations since March? (Check all that apply)

- [x] Yes; Virtually/telemedicine
- [x] Yes; In-person core clerkships
- [x] Yes; In-person sub-internships
- [x] Yes; In-person away rotations
- [x] No; Completely removed from hospitals
- [x] No; Only allowing volunteering opportunities

Which rotations has your medical school offered virtually since March? (Check all that apply)

- [ ] Internal Medicine (and all sub-specialties)
- [ ] Family Medicine
- [ ] Surgery (and all sub-specialties)
- [ ] OB-GYN
- [ ] Pediatrics
- [ ] Neurology
- [ ] Psychiatry
- [ ] Radiology
- [ ] Emergency Medicine
- [ ] Other

If other, please specify:

Which in-person core-clerkships has your medical school offered since March? (Check all that apply)

- [ ] Internal Medicine (and all sub-specialties)
- [ ] Family Medicine
- [ ] Surgery (and all sub-specialties)
- [ ] OB-GYN
- [ ] Pediatrics
- [ ] Neurology
- [ ] Psychiatry
- [ ] Radiology
- [ ] Emergency Medicine
- [ ] Other

If other, please specify:

Which in-person sub-internships has your medical school offered since March? (Check all that apply)

- [ ] Internal Medicine (and all sub-specialties)
- [ ] Family Medicine
- [ ] Surgery (and all sub-specialties)
- [ ] OB-GYN
- [ ] Pediatrics
- [ ] Neurology
- [ ] Psychiatry
- [ ] Radiology
- [ ] Emergency Medicine
- [ ] Other

If other, please specify:

Which in-person away rotations has your medical school offered since March? (Check all that apply)

- [ ] Internal Medicine (and all sub-specialties)
- [ ] Family Medicine
- [ ] Surgery (and all sub-specialties)
- [ ] OB-GYN
- [ ] Pediatrics
- [ ] Neurology
- [ ] Psychiatry
- [ ] Radiology
- [ ] Emergency Medicine
- [ ] Other

If other, please specify:

Has your anticipated medical school graduation year changed due to the coronavirus outbreak?

- ○ Yes; Graduating earlier than expected
- ○ Yes; Graduating later than expected
- ○ No
- ○ Uncertain

Have you participated in in-person clinical rotations during the COVID-pandemic?

- Yes
- No

How well would you say you were briefed on protecting yourself to conduct a rotation at the time of the COVID-pandemic ?

- Very poorly
- Poorly
- Moderately
- Well
- Very well

How well were you equipped (PPE,...) to conduct a rotation at the time of the COVID-pandemic?

- Very poorly
- Poorly
- Moderately
- Well
- Very well

Are you interested in participating in clinical rotations during the coronavirus outbreak?

- Not at all
- Somewhat
- Interested
- Very interested

Which of these, if any, impact your willingness to participate in a clinical rotation or to volunteer at the hospital during the coronavirus pandemic? (Check all that apply)

- [ ] The possibility of being infected myself
- [ ] The possibility of being infected and transmitting to those I live with
- [ ] My lack of social distancing affecting patients in the hospital
- [ ] Concern of using limited PPE
- [ ] Concern of not being an integral member of the team
- [ ] Concern of my medical school's administration judging me if I don't participate
- [ ] Concern of my peers judging me if I don't participate
- [ ] Concern that lack of willingness to volunteer or participate in clinical rotations during the outbreak will impact my evaluations
- [ ] Other

If other, please describe:

Are you experiencing additional challenges/ stressors beyond the normal? (Check all that apply)

- [ ] Academic
- [ ] Financial
- [ ] Mental health
- [ ] Housing stability
- [ ] Health concerns
- [ ] Board exams scheduling
- [ ] Adequate exposure to subspecialties to make career decisions

Over the last two weeks, how often have you been bothered by feeling nervous, anxious or on edge?

- ○ Nearly every day
- ○ More than half of the days
- ○ Several days
- ○ Not at all

Over the last two weeks, how often have you been bothered by feeling down, depressed, or hopeless?

- ○ Nearly every day
- ○ More than half of the days
- ○ Several days
- ○ Not at all

Over the last two weeks, how often have you been bothered by not being able to stop or control worrying?

- ◯ Nearly every day
- ◯ More than half of the days
- ◯ Several days
- ◯ Not at all

Over the last two weeks, how often have you been bothered by little interest or pleasure in doing things?

- ◯ Nearly every day
- ◯ More than half of the days
- ◯ Several days
- ◯ Not at all

Finally, can you provide the following information in order to provide context for your answers?

Age

Sex

- Male-identifying
- Female-identifying
- Non-binary
- Other

In which state do you attend medical school?

- AL - ALABAMA
- AK - ALASKA
- AZ - ARIZONA
- AR - ARKANSAS
- CA - CALIFORNIA
- CO - COLORADO
- CT - CONNECTICUT
- DC - DISTRICT OF COLUMBIA / WASHINGTON DC
- DE - DELAWARE
- FL - FLORIDA
- GA - GEORGIA
- GU - GUAM
- HI - HAWAII
- ID - IDAHO
- IL - ILLINOIS
- IN - INDIANA
- IA - IOWA
- KS - KANSAS
- KY - KENTUCKY
- LA - LOUISIANA
- ME - MAINE
- MD - MARYLAND
- MA - MASSACHUSETTS
- MI - MICHIGAN
- MN - MINNESOTA
- MS - MISSISSIPPI
- MO - MISSOURI
- MT - MONTANA
- NE - NEBRASKA
- NV - NEVADA
- NH - NEW HAMPSHIRE
- NJ - NEW JERSEY
- NM - NEW MEXICO
- NY - NEW YORK
- NC - NORTH CAROLINA
- ND - NORTH DAKOTA
- OH - OHIO
- OK - OKLAHOMA
- OR - OREGON
- PA - PENNSYLVANIA
- PR - PUERTO RICO
- RI - RHODE ISLAND
- SC - SOUTH CAROLINA
- SD - SOUTH DAKOTA
- TN - TENNESSEE
- TX - TEXAS
- UT - UTAH
- VT - VERMONT
- VA - VIRGINIA
- VI - VIRGIN ISLANDS
- WA - WASHINGTON
- WV - WEST VIRGINIA
- WI - WISCONSIN
- WY - WYOMING
